# Evaluation of parameters used in echocardiography and ultrasound protocol for the diagnosis of shock etiology in emergency setting

**DOI:** 10.1186/s12873-023-00902-x

**Published:** 2023-11-09

**Authors:** Asmaa Ramadan, Tamer Abdallah, Hassan Abdelsalam, Ahmed Mokhtar, Assem Abdel Razek

**Affiliations:** 1 Department of Emergency Medicine, Alexandria University, Alexandria, Egypt; 2 Department of Critical Care Medicine, Alexandria University, Alexandria, Egypt; 3 Department of Radiology, Alexandria University, Alexandria, Egypt; 4 Department of Cardiology and Angiology, Alexandria University, Alexandria, Egypt; 5 Department of Anesthesia and Surgical Intensive Care Unit, Alexandria University, Alexandria, Egypt

**Keywords:** Echocardiography, Ultrasound, Shock

## Abstract

**Background:**

Early recognition and appropriate treatment has shown to decrease morbidity and mortality in patients with undifferentiated shock. There are many ultrasound protocols in shock; each protocol combines core ultrasound elements such as IVC and cardiac assessment which includes detection of cardiac tamponade, left ventricular function and right ventricular strain.Valvular assessment is absent in majority of ultasound protocols, while lung ultrasound is included in some of them.

**Objective:**

In this study we investigated which parameters used in Echo – US protocol help differentiate shock types.

**Methods:**

This cross sectional study was conducted on 150 patients with shock (140 patients were included while, 10 patients were excluded).Sensitivity and specificity of different parameters used in the Echo-US protocol were analyzed to detect which parameters can diffentiate shock types.

**Results:**

Velocity time integral of Aorta and IVC maximum diameter were good discriminators for distributive shock, with area under the ROC curve (AUC) = 0.8885 (95% CI 0.8144 to 0.9406) and 0.7728 (95% CI 0.6832 to 0.8473) (Z = 10.256 *p* < 0.0001) and (Z = 5.079 *p* < 0.0001) respectively. Left ventricular systolic function, presence of pneumonia, pneumothorax or valve vegetations were of great value in differentiating shock types, while CUST, FAST, TAPSE and RV diameter were not useful in differentiating shock types.

**Conclusion:**

Ultrasound and echocardiography are powerful tools that can be used to identify shock etiology when the clinical picture overlaps.

## Background

Shock is a circulatory failure that results in inadequate oxygen delivery to tissues [1]. This insufficient perfusion impairs oxidative metabolism and leads to cell death, however, shock is reversible if prompt diagnoses and rapid initiation of specific treatment are done [2]. Previous studies have reported mortality rates ranging from 16 to 25% among cases with non-traumatic undifferentiated shock. These poor outcomes underline the importance of utilizing a systematic approach to identify shock etiology [3]. Early appropriate management aimed at avoiding prolonged hypotension has shown to decrease morbidity and mortality in patients with undifferentiated shock. The use of point of care ultrasound (POUS) has improved diagnostic accuracy from 60.6% to 85.0% for those patients [4]. Different ultrasound protocols for shock assessment have been described since 2001 such as: Focused Assessed Transthoracic Echocardiography (FATE) protocol, which assesses left ventricle contractility, chamber dimension and pleural pathology [5], Focused Echocardiographic Evaluation in Life Support and Peri- Resuscitation (FEEL) study, which is applied to those in cardiac arrest to distinguish between two forms of pulseless electrical activity [6], and FALLS protocol (Fluid Administration Limited by Lung Sonography) to rule out presence of cardiac tamponade, tension pneumothorax, pulmonary embolism and pulmonary edema [7]. One of the most important protocols in shock is the RUSH protocol which involves three parts simplified as pump, tank and pipes [8]. Heart as the pump, the tank (IVC, internal jugular veins (IJV), lungs, pleural cavity, and peritoneal cavity) and the pipes ( aorta, femoral veins and popliteal veins) [9]. Although, most of these protocols combine core ultrasound elements as inferior vena cava (IVC) and cardiac assessment, valvular assessment is absent in most ultasound protocols while lung ultrasound is included in only some of them [8]. Hitherto, None of these ultrasound protocols has investigated the senstivity and specificity of different parameters used in each of them to determine whether or not these parameters help to identify shock etiology.

The aim of this study is to investigate which echocardiography and ultrasound parameters help to differentiate various types of shock.

## Material and methods

This cross sectional study was performed prospectively at the Emergency Department of Alexandria Main University Hospital—a tertiary care center in the North of Egypt- between December 2018 and December 2020.

The study was conducted on a convenience sample of 150 patients with shock admitted during the clinical shifts of the study conductor (36 h per week). All patients with shock who met the inclusion criteria of this study were enrolled, including obese patients or patients with hyper inflated chest with no selection bias. Shock was defined as SBP < 90 mmHg or shock index ≥ 1 with evidence of tissue hypo perfusion indicated by presence of delayed capillary refill time, altered mental state, low urine output, mottled skin and/or elevated lactate level [10]. Exclusion criteria were patients with trauma, post cardiac arrest, burn, arrhythmias or those who received fluids prior to hospital admission.

All ultrasound examinations were carried out by the first author, who is Point of Care Ultrasound (POCUS) & Egyptian Medical Society of Echocardiography certified with 5 years’ experience in emergency ultrasound. The study was approved by Alexandria University Ethical Committee (Reference number 0201184). Informed consent was taken from all participants or their next of kin.

Echocardiography was done using Vivid e-machine (General Electric, Boston, USA) with 2.8–4 MHz phased array probe in long, short axis parasternal view, RV inflow and outflow tract, apical three, four or five chamber view. Different alternatives were used as some views could not be visualized in all patients. Ultrasound was performed by the study conductor parallel to initial assessment and resuscitation of patients which were done by treating physicians without interruption or delay of patient care. Examination was initiated with echocardiography to assess left ventricular outflow tract velocity time integral (LVOT VTI), IVC maximum diameter, collapsibility or distensibility index, which was conducted before any fluid were administered. For accurate measurement of LVOT VTI, the pulsed wave marker was placed directly in line with LVOT blood flow and the pulsed wave sampling gate was placed at LVOT above the Aortic valve [11]. IVC collapsibility index was measured in normal respiration in case of spontaneously breathing patients; while IVC distensibility index was calculated in mechanically ventilated patients. Other echo parameters assessed were: left ventricular (LV) systolic function, presence of pericardial effusion, tricuspid annular plane systolic excursion (TAPSE), right ventricular outflow tract (RVOT) diameter, valve vegetation, and ruptured aortic aneurysm. Each measurement of Echo- US protocol was performed once because if more than one measurement was taken this would prolong the scan time. Ultrasound was performed by one of three devices: Mindray DC-30, DP-5 and DP-20 (Mindray, Shenzhen, China) with convex and linear probes to detect presence of pneumonia, pleural effusion, pneumothorax, deep vein thrombosis and intra-abdominal collection.

Classification of shock types was made based on the following:

- Hypovolemic/ distributive shock:

Both types of shock were identified by presence of: hyper dynamic heart, small collapsible IVC, normal RV diameter and function while, the presence of high LVOT VTI, empyema, pneumonia or infective endocarditis helped to distinguish distributive from hypovolemic shock.

- Cardiogenic shock:

In this type of shock there was reduced LV function, valvular lesion, low VTI, large non- collapsible IVC and diffuse B- lines throughout the lung.

- Obstructive shock:

Diagnosis was made when signs of pneumothorax, cardiac tamponade or acute massive pulmonary embolism were found.

- Mixed distributive- cardiogenic shock:

This was suggested when discordant findings were detected in patients with distributive shock (low VTI and reduced LV systolic function).

- Mixed distributive -obstructive:

Completing the scan revealed high VTI or ultrasound findings of pneumonia which raised the concern of an underlying septic focus.

Patients received all required diagnostic and therapeutic interventions by the treating emergency physicians without delay and were followed to document their final diagnosis. Ultrasound protocol findings were correlated to reach an initial impression about shock type but not final diagnosis. A second physician established the final diagnosis, other than the emergency physician, to whom the patient was transferred to Intensive Care unit, Cardiology Department, Internal Medicine Unit or Surgical Unit. Final diagnosis was made according to reference standard for each shock type as: echocardiography performed by cardiologist for massive pulmonary embolism, cardiac tamponade and cardiogenic shock, laboratory investigations and radiological imaging including CT chest, ultrasound abdomen, CT abdomen and pelvis, interpreted by certified radiologist for septic shock. Treating physicians were not blind to ultrasound exam findings where ultrasound protocol identified life threatening conditions (39.3%) of cases such as: cardiac tamponade, mechanical complication of acute myocardial infarction, pulmonary embolism and mixed shock etiology, which would otherwise have been missed.

Sensitivity and specificity of different parameters used in the Echo-US protocol were analyzed to detect which parameters can differentiate shock types.

### Data analysis

- Data were collected and entered to the computer using SPSS program (IBM company, Chicago, USA) for statistical analysis (version 21) [12]. Data were entered as numerical or categorical, as appropriate. Kolmogorov-Smirnov test of normality revealed significance in the distribution of the variables, so the non-parametric statistics was adopted [13]. Diagnostic test evaluation was carried out using MedCalc Software version 14 [14]. Two by two tables were constructed and sensitivity, specificity, positive predictive value (PPV), negative predictive value (NPV) and accuracy were calculated. Area under the ROC (AUC) was carried out using MedCalc Software version 14. The Youden index was used to determine the best cut-off value [15]. Based on previous studies, the prevalence of cardiogenic shock was 26% [16]. A minimum required sample was 135 patients with shock to detect sensitivity (90%) of focused echo and ultrasound protocol, using precision of 10% with alpha error of 0.05. This calculation was based on Burderer^,^ s formula for sensitivity and specificity of diagnostic health studies. To consider and anticipate drop rate of 10%, the sample size was increased to include150 patients with shock.

## Results

We recruited 140 patients including 71 males (50.7%) and 69 female (49.3%) as in Table 1, where10 patients were excluded (6 patients died while, 4 patients discharged against medical advice before reaching final diagnosis). The median age was 60 years (Interquartile range, IQR, 47–73). The median systolic blood pressure (SBP) was 80 mmHg. Thirty six cases had unrecorded SBP by noninvasive method. Diagnosis of shock in these cases depended on presence of tachycardia and indicators of tissue hypo perfusion.

**Table 1:** Demographic and clinical characteristics of the studied subjects

	**Number**	**Min–max**	**Median**	**Interquartile range**
Sex	140	71 males/ 69 females	-----	-----
Age (years)	140	24 -96	60	47–73
Systolic blood pressure (mmHg)	104	50–110	80	70–90
Diastolic blood pressure (mmHg)	102	30–70	50	40–50
Mean blood pressure (mmHg)	102	36.7–83.3	60	50–63.3
Heart rate (bpm)	140	40–150	100	90–120
Initial lactate (mmol/L)	140	0.20 – 15	3.55	2.15–6.50

Some ultrasound and echo parameters were not feasible in all patients as shown in Tables 2 and 3. IVC and aorta were not seen in 10% and 17% of cases respectively, due to presence of bowel gases and adiposity also; RVOT diameter and TAPSE were unmeasured in approximately 18% of patients due to inability to get long axis parasternal and apical four-chamber views. One patient had a large femoral abscess on the thigh which precluded performing compression using ultrasound test (CUST) test. Despite this, we still achieved a high completion rate during the examination. Senstivity and specificity of different Echo and US parameters (Tables 2 and 3) showed that reduced LV function was highly sensitive for cardiogenic and mixed distributive cardiogenic shock 91.67% and 100% (95% CI 61.52% to 99.79% and 61.52% to 99.79%) respectively. Pericardial effusion was present in 12.14% of cases, 41.2% of them had distributive shock as shown in Table 4. Cardiac tamponade was present only in cases of mixed distributive- obstructive shock with sensitivity, specificity and positive likelihood ratio of 100%, 91.11% and 11.25 respectively.

**Table 2:** Sensitivity of different Echo-US parameters in each shock type at 95% confidence interval (CI)

Parameter (Number of cases measured)	Distributive	Cardiogenic	Hypovolemic	Obstructive	Mixed distributive cardiogenic	Mixed distributive obstructive
Pericardial effusion (140)	**7 (6.12%)** 2.28% to 12.85%	**1 (8.33%)** 0.21% to 38.48%	**1 (14.29%)** 0.36% to 57.87%	**1 (25%)** 0.63% to 80.59%	**2 (21.43%)** 4.66% to 50.80%	5 (100%^*^) 47.82% to 100%
RVOT diameter > 3 cm (114)	**59 (73.75%)** 62.71% to 82.96%	**8 (72.73%)** 39.03% to 93.98%	**4 (80%)** 28.36% to 99.49%	3 (100%^*^) 29.24% to 100%	**8 (72.73%)** 39.03% to 93.98%	**3 (75%)** 19.41% to 99.37%
TAPSE < 17 mm (113)	**26 (32.10%)** 22.15% to 43.40%	**5 (50%)** 18.71% to 81.29%	**1 (25%)** 0.63% to 80.59%	3 (100%^*^) 39.76% to 100%	**7 (63.64%)** 30.79% to 89.07%	**1 (33.33%)** 0.84% to 90.57%
Reduced LV function (140)	**8 (8.16%)** 3.59% to 15.45%	11 (91.67%^*^) 61.52% to 99.79%	**0 (0%)** 0% to 40.96%	**0 (0%)** 0% to 60.24%	14 (100% *) 76.84% to 100%	**0 (0%)** 0% to 52.18%
Valve vegetations (140)	**3 (3.06%)** 0.64% to 8.69%	**0 (0%)** 0% to 26.46%	**0 (0%)** 0% to 40.96%	**0 (0%)** 0% to 60.24%	**0 (0%)** 0% to 23.16%	**0 (0%)** 0% to 52.18%
Tension Pneumothorax (140)	**0 (0%)** 0% to 3.69%	**0 (0%)** 0% to 26.46%	**0 (0%)** 0% to 40.96%	**0 (0%)** 0% to 60.24%	**0 (0%)** 0% to 23.16%	3 (60%^*^) 14.66% to 94.73%
Positive CUST (139)	**12 (12.37%)** 6.56% to 20.61%	**1 (8.33%)** 0.21% to 38.48%	**1 (14.29%)** 0.36% to 57.87%	**0 (0%)** 0% to 60.24%	**1 (7.14%)** 0.18% to 33.87%	**0 (0%)** 0% to 52.18%
Pneumonia (140)	**33 (33.67%)** 24.44% to 43.93%	**0 (0%)** 0% to 26.46%	**0 (0%)** 0% to 40.96%	**0 (0%)** 0% to 60.24%	10 (71.43%^*^) 41.90% to 91.61%	4 (80%^*^) 28.36% to 99.49%
Positive FAST (140)	**22 (22.45%)** 14.64% to 31.99%	**3 (25%)** 5.49% to 57.19%	**5 (71.43%)** 29.04% to 96.33%	**2 (50%)** 6.76% to 93.24%	**4 (28.57%)** 8.39% to 58.10%	**2 (40%)** 5.27% to 85.34%
Pleural effusion (140)	**17 (17.35%)** 10.44% to 26.31%	**1 (8.33%)** 0.21% to 38.48%	**0 (0%)** 0% to 40.96%	**0 (0%)** 0% to 60.24%	**6 (42.86%)** 17.66% to 71.14%	4 (80%^*^) 28.36% to 99.49%
AAA (116)	**0 (0%)** 0% to 3.69%	**0 (0%)** 0% to 26.46%	**2 (28.57%)** 3.67% to 70.96%	**0 (0%)** 0% to 60.24%	**0 (0%)** 0% to 23.16%	**0 (0%)** 0% to 52.18%

*RVOT* Right ventricular outflow tract, *TAPSE* Tricuspid annular plane systolic excursion, *LV* Left ventricle, *CUST* Compression using ultrasound test, *FAST* Focused assessment with sonography for trauma, *AAA* Abdominal aortic aneurysm. *significant

**Table 3:** Specificity of different Echo-US parameters in each shock type at 95% confidence interval

Parameter(Number of cases measured)	Distributive	Cardiogenic	Hypovolemic	Obstructive	Mixed distributive cardiogenic	Mixed distributive obstructive
Pericardial effusion (140)	**7 (73.81%)** 57.96% to 86.14%	**1 (87.50%)** 80.50% to 92.68%	**1 (87.97%)** 81.20% to 92.96%	**1 (88.24%)** 81.60% to 93.12%	**2 (88.89%)** 82.06% to 93.79%	**5 (91.11%)** 84.99% to 95.32%
RV diameter > 3 cm (114)	**59 (23.53%)** 10.75% to 41.17%	**8 (25.24%)** 17.20% to 34.76%	**4 (25.69%)** 17.80% to 34.94%	**3 (26.13%)** 18.25% to 35.32%	**8 (25.24%)** 17.20% to 34.76%	**3 (25.45%)** 17.63% to 34.65%
TAPSE < 17 mm (113)	**26 (43.75%)** 26.36% to 62.34%	**5 (62.14%)** 52.04% to 71.51%	**1 (60.55%)** 50.73% to 69.78%	**3 (63.30%)** 53.53% to 72.33%	**7 (63.73%)** 53.61% to 73.02%	**1 (55.21%)** 44.71% to 65.37%
Reduced LV function (140)	**8 (40.48%)** 25.63% to 56.72%	**11 (82.81%)** 75.14% to 88.90%	**0 (75.19%)** 66.96% to 82.26%	**0 (75.74%)** 67.64% to 82.67%	**14 (84.92%)** 77.46% to 90.67%	**0 (75.56%)** 67.42% to 82.54%
Valve vegetations (140)	**3 (100%**^a^**)** 91.59% to 100.00%	**0 (97.66%)** 93.30% to 99.51%	**0 (97.74%)** 93.55% to 99.53%	**0 (97.79%)** 93.69% to 99.54%	**0 (97.62%)** 93.20% to 99.51%	**0 (97.78%)** 93.64% to 99.54%
Tension Pneumothorax (140)	**0 (92.86%)** 80.52% to 98.50%	**0 (97.66%)** 93.30% to 99.51%	**0 (97.74%)** 93.55% to 99.53%	**0 (97.79%)** 93.69% to 99.54%	**0 (97.62%)** 93.20% to 99.51%	**3 (100%**^a^**)** 97.30% to 100.00%
Positive CUST (139)	**12 (92.86%)** 80.52% to 98.50%	**1 (88.98%)** 82.20% to 93.84%	**1 (89.39%)** 82.85% to 94.08%	**0 (88.89%)** 82.34% to 93.65%	**1 (88.80%)** 81.92% to 93.74%	**0 (88.81%)** 82.21% to 93.60%
Pneumonia (140)	**33 (66.67%)** 50.45% to 80.43%	**0(63.28%)** 54.31% to 71.62%	**0 (64.66%)** 55.91% to 72.75%	**0 (65.44%)** 56.81% to 73.38%	**10 (70.63%)** 61.86% to 78.41%	**4 (68.15%)** 59.58% to 75.90%
Positive FAST(140)	**22 (61.90%)** 45.64% to 76.43%	**3 (72.66%)** 64.08% to 80.16%	**5 (75.19%)** 66.96% to 82.26%	**2 (73.53%)** 65.28% to 80.72%	**4 (73.02%)** 64.38% to 80.53%	**2 (73.33%)** 65.04% to 80.57%
Pleural effusion (140)	**17 (73.81%)** 57.96% to 86.14%	**1 (78.91%)** 70.81% to 85.62%	**0 (78.95%)** 71.03% to 85.53%	**0 (79.41%)** 71.64% to 85.86%	**6 (82.54%)** 74.77% to 88.72%	**4 (82.22%)** 74.71% to 88.26%
AAA (116)	**0 (95.24%)** 83.84% to 99.42%	**0 (98.44%)** 94.47% to 99.81%	**2 (100%**^a^**)** 97.26% to 100%	**0 (98.53%)** 94.79% to 99.82%	**0 (98.41%)** 94.38% to 99.81%	**0 (98.52%)** 94.75% to 99.82%

^a^significant

**Table 4:** Distribution of studied cases according to pericardial effusion (tamponading or not)

**Tamponade**	**Final shock type**						**Total**
	**Distributive**	**Cardiogenic**	**Hypovolemic**	**Obstructive**	**Mixed distributive cardiogenic**	**Mixed distributive obstructive**	
**No tamponade (*n* = 14)**							
- n	7	1	1	0	2	3	14
- % within tamponade	50.00%	7.14%	7.14%	0.00%	14.29%	21.43%	82.35%
- % within final shock type	100.00%	100.00%	100.00%	0.00%	100.00%	60.00%	
**Tamponade (*n* = 3)**							
- n	0	0	0	1	0	2	3
- % within tamponade	0.00%	0.00%	0.00%	33.33%	0.00%	66.67%	17.65%
- % within final shock type	0.00%	0.00%	0.00%	100.00%	0.00%	40.00%	
**Total (*n* = 17)**							
- n	7	1	1	1	2	5	17
- %	41.18%	5.88%	5.88%	5.88%	11.76%	29.41%	100.00%
**Test of significance** **(*p* value)**	χ^2^_(df=5)_ = 8.743 *p*_(MC)_ = 0.143 NS						

*n* Number of specimens, *MC* Monte Carlo correction for *p* value of Pearson Chi square test, *df* Degree of freedom, *NS* Statistically not significant (*p ≥ *0.05)

Obstructive shock was caused by massive pulmonary embolism (PE) in 75% of cases while, 25% had cardiac tamponade. RVOT diameter > 3 cm and TAPSE < 17 mm were 100% sensitive for cases of acute massive pulmonary embolism, but with low specificity 26.13% (95% CI 18.25% to 35.32%) and 63.30% (95% CI 53.53% to 72.33%) respectively.

**Fig. 1:**
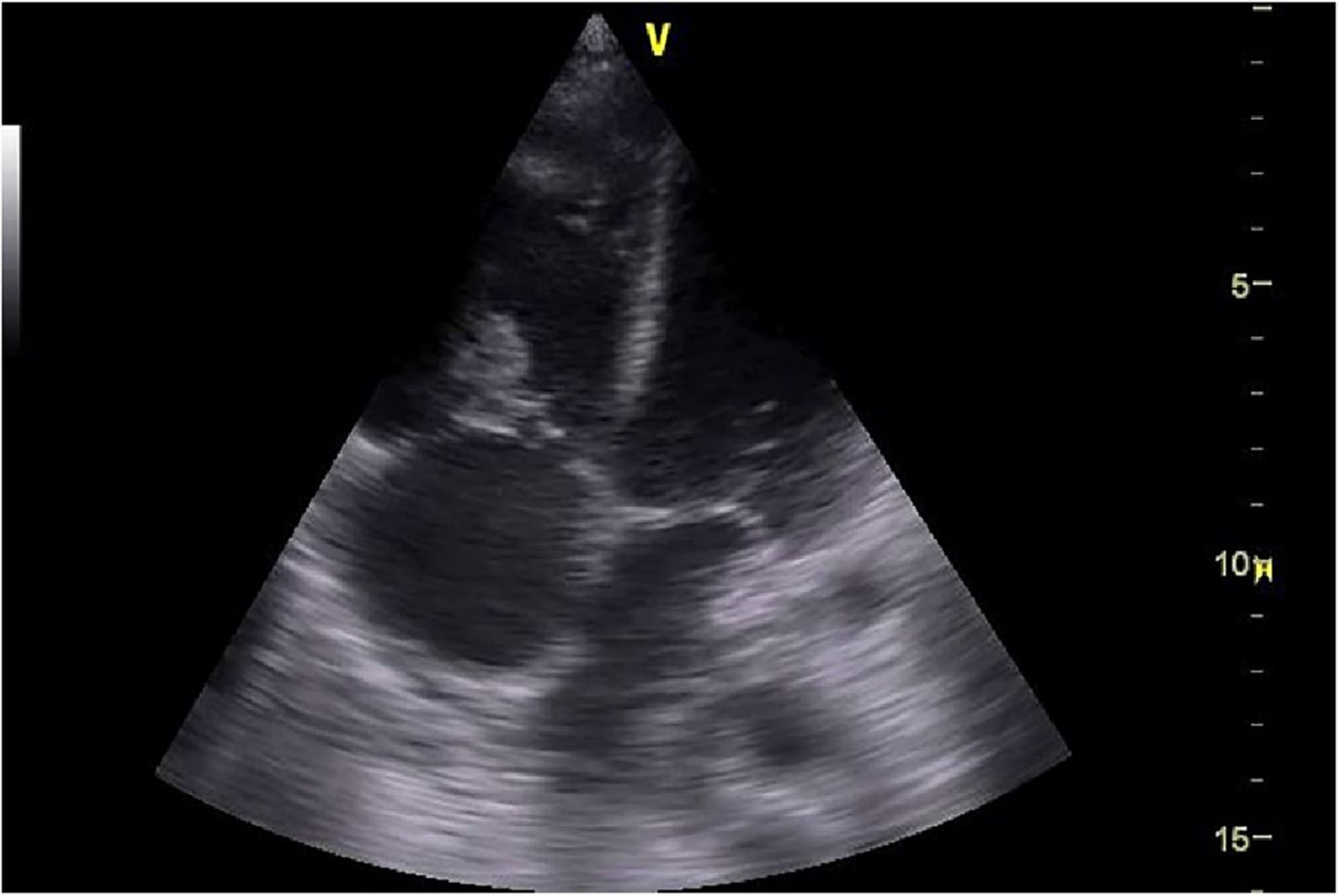
Apical four chamber view showing vegetation on tricuspid valve

**Fig. 2:**
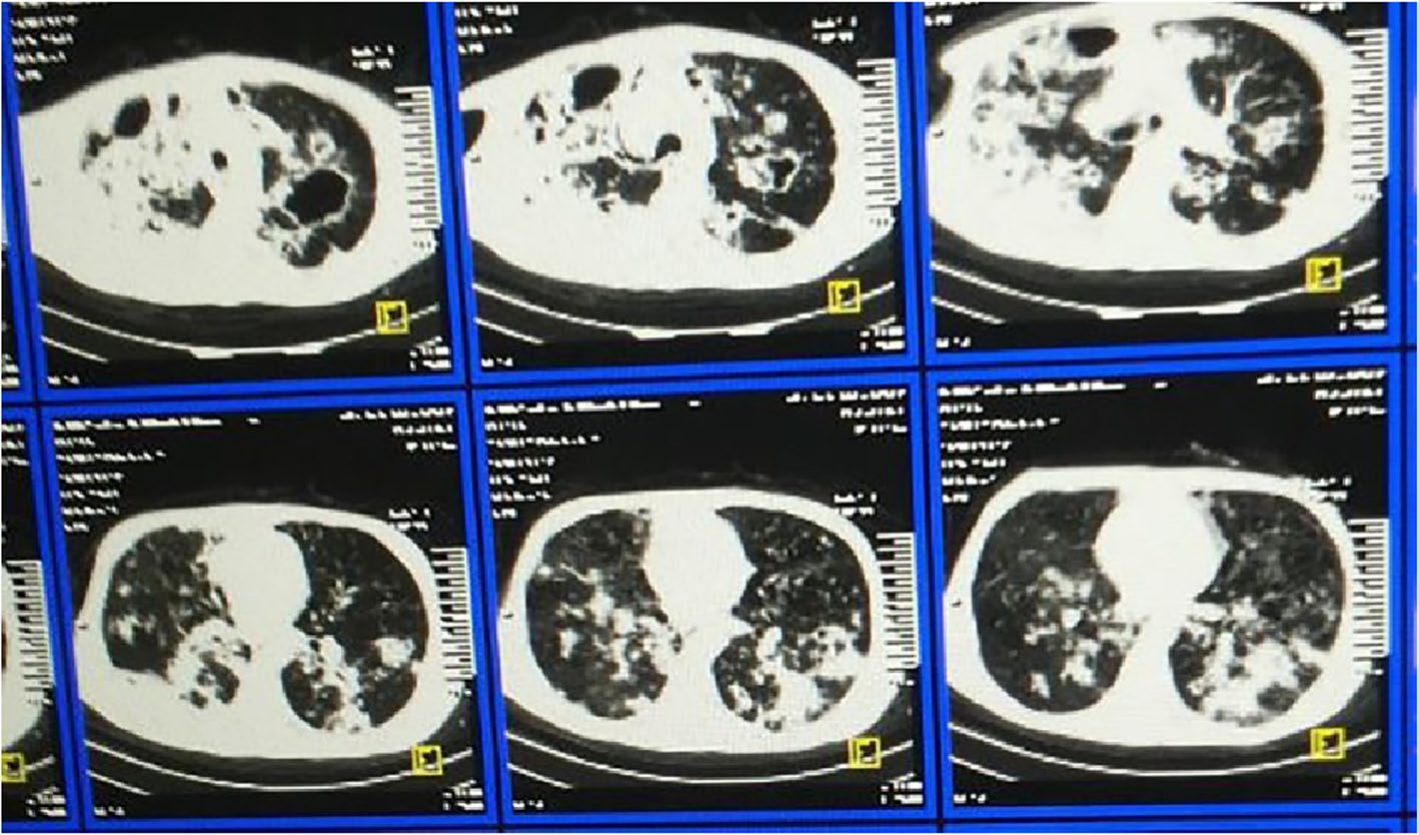
CT chest shows septic emboli in patient with tricuspid infective endocarditis

Although tricuspid infective endocarditis had low sensitivity for distributive shock (3.06%), it helped to identify the source of sepsis as demonstrated in Figs. 1 and 2. Moreover, pneumothorax was absent in all types of shock except mixed distributive- obstructive shock with sensitivity 60% (95% CI 14.66% to 94.73%). A striking finding was that positive CUST test had 0% sensitivity and positive likelihood ratio for both obstructive and mixed distributive—obstructive shock and 88.8% specificity but, it was positive in all other types of shock with sensitivity between 7.14% and 14.29%.

Ultrasound findings of pneumonia were present in distributive or mixed shock types with sensitivity between 33.67% and 80%. FAST exam could not be used to differentiate shock types as it was positive in all types of shock such as ascites, intraabdominal hemorrhage or from perforated viscus with senstivity ranging from 22.45% to 71.43%.

**Fig. 3:**
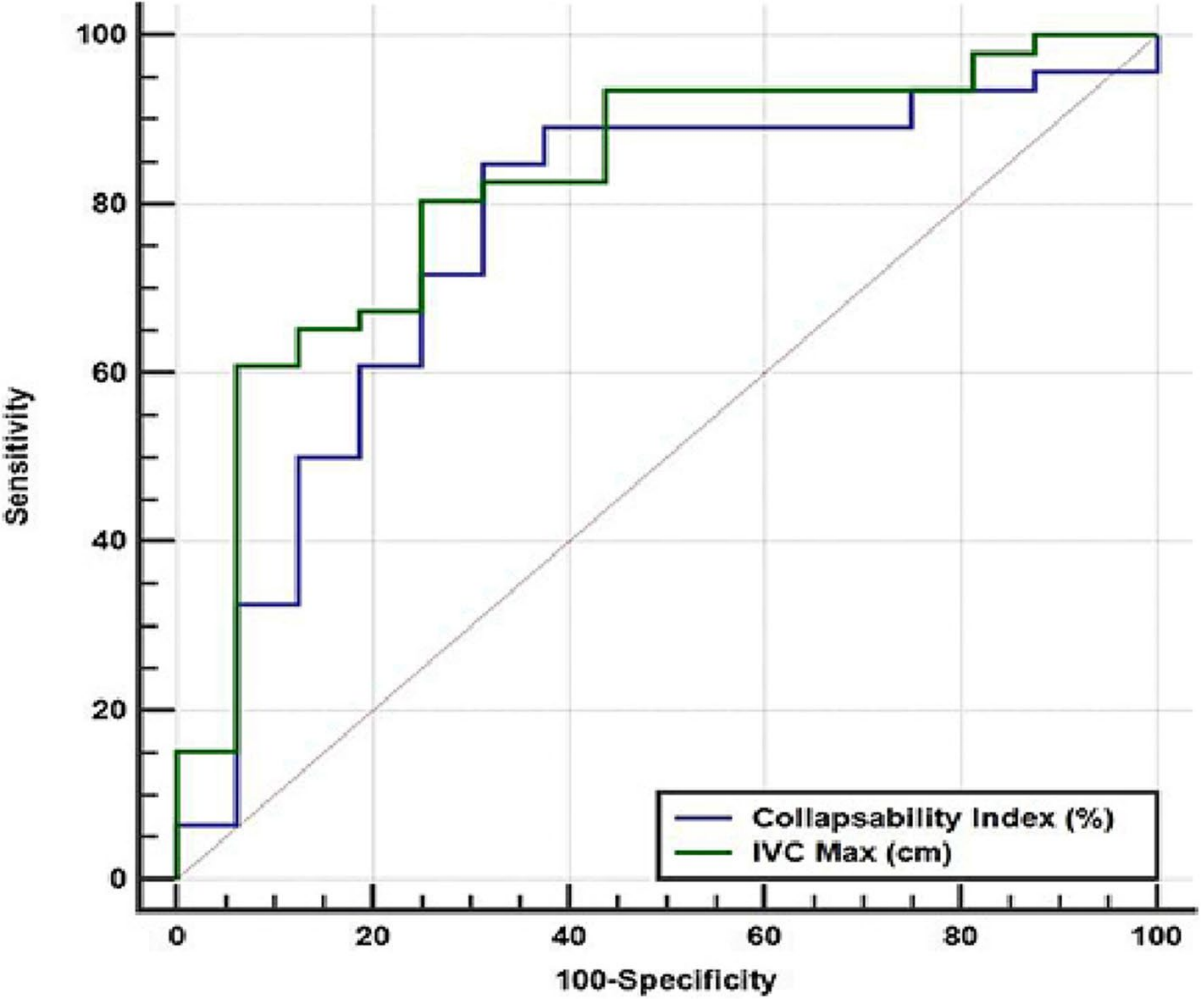
Area under the ROC curve for collapsibility index (%) and IVC max (cm) as discriminator for distributive or hypovolemic shock

Comparison between area under the ROC curve of IVC maximal diameter (cm) and IVC collapsibility index (%) as discriminators of hypovolemic or distributive shock showed that both were good discriminators of these types of shock as compared to other shock types with area under ROC curve (AUC) = 0.8207 (95% CI 0.7025 to 0.9065) and 0.7622 (95% CI 0.6371 to 0.8612) respectively (Fig. 3). Diagnostic criteria using Youden index was ≤ 1.93 cm for IVC maximal diameter with senstivity 80.43% (95% CI 66.1—90.6), specificity 75% (95% CI 47.6- 92.7) PPV 85.9% ( 95% CI 78.2- 91.2) and NPV 61.5% (95% CI 49.2–72.6%). IVCCI > 25.5% had sensitivity 84.78% (95% CI 71.1–93.7), specificity 68.75% (95% CI 41.3–89), PPV 88.6% (95% CI 78.9–94.2%) and NPV 62.2% (95% CI 50.9–72.3). IVC collapsibility index at 50% had sensitivity 36.96% (95% CI 23.2–52.5), specificity 87.5% (95%CI 61.7- 98.4%), PPV 89.5% (95%CI 68.8–97%) and NPV 32.6% (95%CI 26.6–39.2) for diagnosis of hypovolemic or distributive shock.

**Fig. 4:**
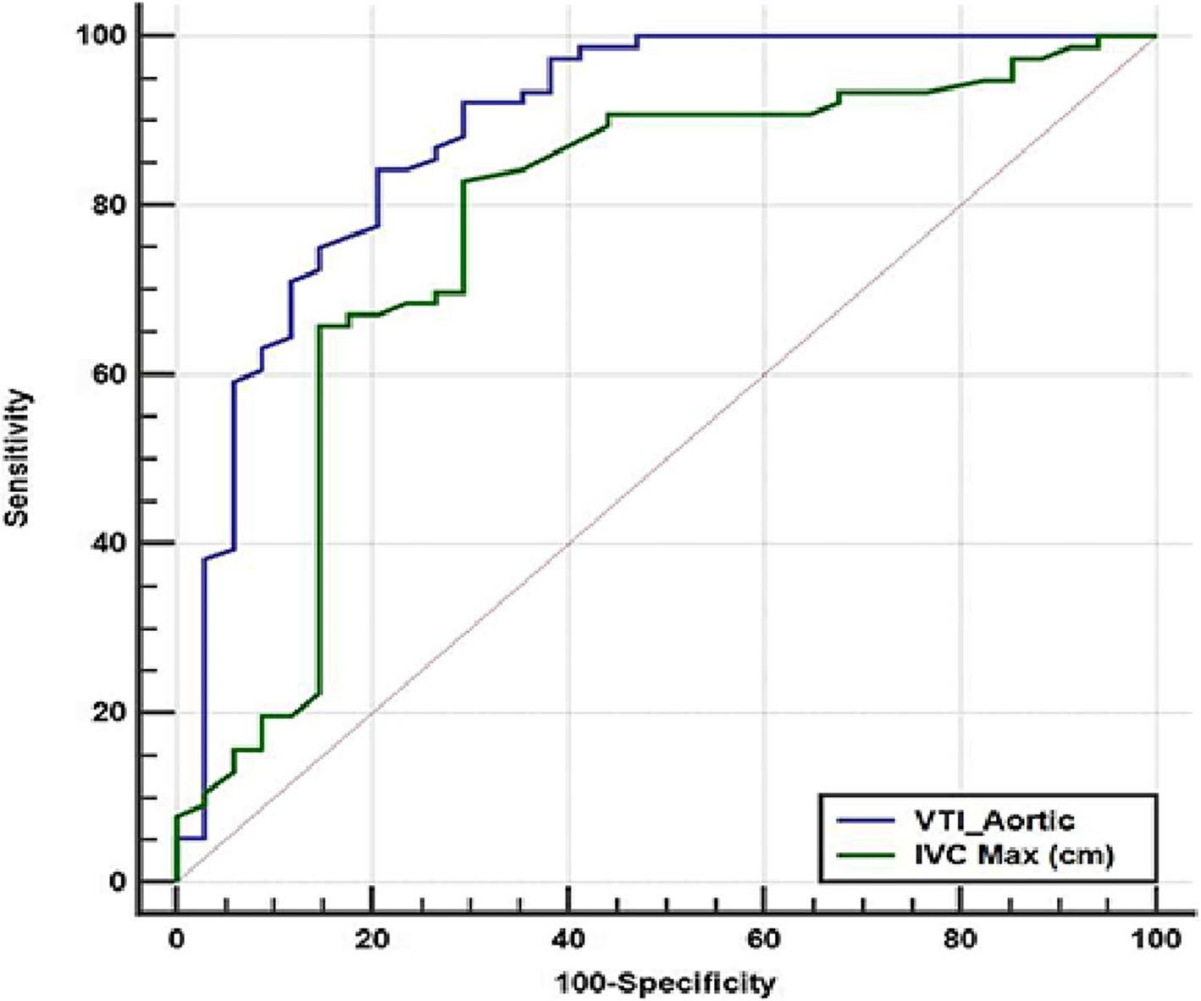
Area under the ROC curve for VTI aortic (cm) and IVC max (cm) as discriminator for distributive shock

Comparison between area under the ROC curve for VTI aorta (cm)versus IVC maximum diameter (cm) as discriminators for distributive shock demonstrated that both were good discriminators for distributive shock with area under the ROC curve (AUC) = 0.8885 (95% CI 0.8144 to 0.9406) and 0.7728 (95% CI 0.6832 to 0.8473) (Z = 10.256 *p* < 0.0001) and (Z = 5.079 *p* < 0.0001) respectively (Fig. 4). The diagnostic criteria using Youden index for VTI aorta > 15.6 cm had senstivity 84.21% (95% CI 74 – 91.6), specificity 79.41% ( 95% CI 62.1–91.3), PPV 90.1% (95% CI 82.4–94.7) and NPPV 69.2% ( 95% CI 56.6–79.5) for diagnosis of distributive shock. IVC maximal diameter ≤ 2.01 cm had senstivity 82.89% ( 95% CI 72.5 – 90.6), specificity 70.59% (95% CI 52.5–84.9), PPV 86.3% (95% CI 78.7—91.5) and NPV 64.9% (51.8–76) for diagnosis of distributive shock.

## Discussion

In the emergency setting, clinicians perform ultrasound to address the etiology of shock before advanced mointoring and investigations are available. In this study we have found that not all parameters of echocardiography and ultrasound were of value in differentiating shock types. IVC maximal diameter and VTI Aorta were significant discriminator for distributive shock. Other parameters were present significantly in certain types of shock such as pneumonia in distributive or mixed shock types and reduced LV function in cardiogenic and mixed distributive cardiogenic shock. CUST, FAST and RV dysfuction were positive nearly in all types of shock.

IVC maximal diameter ≤ 1.93 cm was a good discriminator of hypovolemic or distributive shock with area under ROC curve (AUC) = 0.8207. Atkinson et al. found that IVC maximum diameter < 2 cm with collapsibility index > 40–50% indicates hypovolemic or distributive shock [17]. The American Society of Echocardiography suggested IVC diameter < 2.1 cm with > 50% collapsibility index correlates with right atrial pressure (0–5 mm Hg), while an IVC diameter > 2.1 cm with collapsibility index < 50% suggests right atrial pressure (10–20 mmHg) [18].

In this study IVCCI > 25.5% had a sensitivity of 84.78%, specificity of 68.75%, PPV 88.6% and NPV62.2%, while Mok et al. [18] showed that IVCCI using cut off 40% had sensitivity 70%, specificity 80%, PPV 72% and NPV 83% for detection of hypovolemic or distributive shock. Some patients had tricuspid regurgitation, pericardial effusion, elevated intraabdominal pressure or preexisting reduced left ventricular function which contributed to a large but non- collapsible IVC despite having hypovolemic or distributive shock.

Blanco et al. [11] showed that LVOTVTI > 18 cm is a key characteristic finding in distributive shock which was correlated with high cardiac output in this type of shock. In this study LVOT VTI > 15.6 was a good discriminator for distributive shock with area under the ROC curve (AUC) = 0.8885 with senstivity 84.21% and specificity 79.41%. Some patients with distributive shock had severe hypovolemia; others had reduced LV function which resulted in low VTI.

It is essential to look for evidence of pericardial effusion in the context of critically ill patients, but it is more important to look for presence of tamponade as pericardial effusion not causing tamponade, can be present in any shock type. In this study, pericardial effusion was present in 17 patients (12.14%) but tamponade was merely present in 3 cases. This was similar to results of study carried out by Zou et al. [19] in which 26 patients with shock (14.4%) had pericardial effusion but only one case had cardiac tamponade. The underlying pathophysiology for this incidental finding in other shock types was malignancy, chronic renal failure, radiotherapy and acute inflammatory pericarditis.

Low specificity for RVOT diameter > 3 cm and TAPSE < 17 mm for diagnosis of acute pulmonary embolism in this study correlated with the study done by Zou et al. [19] in which RV dysfunction was found in 68 shocked cases (47.2%) while acute pulmonary embolism was present only in 3 cases (1.66%). This highlights the importance of combining other echocardiographic signs of acute pulmonary embolism as: D- shaped LV, tricuspid regurgitation, paradoxical septal motion, large non collapsible IVC, McConnells sign and right ventricular wall thickness < 5 mm where some patients had preexisting pulmonary disease leading to pulmonary hypertension or other diseases involving the right ventricle as acute coronary syndrome or sepsis.

Compression using ultrasound test (CUST) was used for detection of deep vein thrombosis in lower limbs. CUST was negative in all cases of acute pulmonary embolism but, it was positive in patients with hypovolemic, cardiogenic and distributive shock in which advanced age, malignancy or prolonged immobilization were the underlying risk factors. This correlates with a study carried out by Atkinson et al. [17] who found that absence of thrombus in lower limb did not rule out acute pulmonary embolism. Moreover, Righini et al. [20] had found that CUST was positive only in 30–50% of patients with pulmonary embolism. It is interesting to note that even when CUST test was positive, causes of shock other than pulmonary embolism could remain a possibility. Distributive shock was the most likely type of shock with positive CUST which had a positive predictive value of 80% (95% CI 54.34- 93.08%) and positive likelihood ratio of 1.73 (95% CI 0.52- 5.82%). Nazerian et al. [21] conducted study on cohort group of patients with shock with suspected acute pulmonary embolism, DVT was confirmed in 27 patients (25.7%) and 88.9% of them had a final diagnosis of PE, whereas 3 patients (11.1%) had diagnosis other than PE. In our study CUST was positive in 15 patients (10.79%) and 80% of them had final diagnosis of distributive shock. Lower number of patients with positive CUST in our study was due to 70% of the included patients had distributive shock, whereas only 2.86% of cases had obstructive shock while, Nazerian et al. conducted the study on (105) patients with suspected acute pulmonary embolism.

The sensitivity of ultrasound findings of pneumonia for detection of distributive, mixed distributive-cardiogenic and mixed distributive-obstructive were 33.67%, 71.43% and 80% respectively. The highest positive predictive value of pneumonia was for distributive shock 70.21% (95% CI 58.60%—79.70%). Pneumonia had similar specificity in all shock subtypes. This could be attributed to 70% of the studied cases had distributive shock, thus when calculating specificity for distributive shock true negative cases constituted about 20% of studied cases versus approximately 63% in other shock types. This agrees with study done by Vaidya et al. [22] in which pneumonia was present only in 20.5% of patients with distributive shock as not all cases with septic shock had chest infection.

Although FAST exam was positive nearly in all types of shock, this provides a clue to septic focus as some cases had perforated abdominal organ or intra- abdominal abscesses that when integrated with other US findings, the diagnosis of septic shock was concluded. Trauma patients were excluded from this study but, three cases with hypovolemic shock had positive FAST exam. One patient had intra-abdominal hemorrhage due to ruptured aneurysm of celiac and renal arteries in a known case of fibromyodysplesia and the other two cases had rupture abdominal aortic aneurysm.

Severe left side valvular disease was one of the causes of cardiogenic shock in this study. These valvular lesions were either a complication of acute myocardial infarction or acute decompensation in setting of underlying severe chronic LV systolic dysfunction. The presence of tricuspid infective endocarditis, despite having low senstivity for detection of distributive shock 3.06%, its specificity was 100%. Moreover, it contributed to the early detection of the septic focus especially for those at high risk for infective endocarditis (Figs. 3 and 4).

More comprehensive echocardiography was needed to diagnose all cases of cardiogenic shock as RUSH exam had a high positive likelihood ratio of 22.29 but only a moderate likelihood ratio of 0.17, so it is not a perfect test to rule out cardiogenic shock.

In this study, we highlighted that specific parameters used in Echo-US protocol helps in differentiating shock types such as lung ultrasound, LV systolic function, valve assessment, presence of tamponade, IVC maximal diameter and LVOT VTI.

## Limitation

Further studies are needed to identify if implementation of Echo- US protocol could be translated into survival benefit. This study was performed by single operator which might affect generalization of results. We cannot comment on inter- rater reliability of parameters used in the protocol. Combining these individual ultrasound and echo findings together and development of scoring system to reach final conclusion about shock etiology could be a direction for future research.

## Conclusion

Ultrasound and echocardiography are powerful tools that can be used to identify shock etiology when the clinical picture overlaps. The following exam components should be done when assessing patients with shock: LV systolic function; presence of tamponade, valve assessment, LVOT VTI, IVC and lung US, which help to detect the underlying shock etiology. Other less important parameters which did not differentiate shock type were FAST scan, CUST test TAPSE and RV diameter.

## Data Availability

The datasets generated and/or analyzed during the current study are not public available due privacy and data safety but are available from the corresponding author on reasonable request.
